# Alightment of Spotted Wing Drosophila (Diptera: Drosophilidae) on Odorless Disks Varying in Color

**DOI:** 10.1093/ee/nvv155

**Published:** 2015-10-16

**Authors:** D. M. Kirkpatrick, P. S. McGhee, S. L. Hermann, L. J. Gut, J. R. Miller

**Affiliations:** Department of Entomology, Michigan State University, East Lansing, MI 48824, (kirkpa42@msu.edu; mcghee@msu.edu; slh@msu.edu; gut@msu.edu; miller20@msu.edu) and

**Keywords:** *Drosophila suzukii*, insect color vision, trap color, background

## Abstract

Methods for trapping spotted wing drosophila, *Drosophila suzukii* (Matsmura) (Diptera: Drosophilidae), have not yet been optimized for detecting this devastating pest of soft-skinned fruits. Here, we report outcomes of choice and no-choice laboratory bioassays quantifying the rates of spotted wing drosophila alightment on 5-cm-diameter sticky disks of various colors, but no fruit odors. Red, purple, and black disks captured the most spotted wing drosophila when presented against a white background. Male and female spotted wing drosophila responded identically in these tests. Significantly more *D. suzukii *were captured on the red and yellow disks than those presenting the corresponding grayscale for that color, proving that *D. suzukii* perceives colors and not just the level of target brightness. Fluorescent red is the best candidate for trap color, while clear and white are the least desirable. However, when the background was switched to black, all nonfluorescent colors were equally acceptable to spotted wing drosophila, suggesting that background must be specified when reporting spotted wing drosophila color preference. Additional spotted wing drosophila research is justified on the effects of target color against natural backgrounds.

Spotted wing drosophila, *Drosophila suzukii *(Matsumura) (Diptera: Drosophilidae), is a recently introduced and highly invasive pest in the United States that destroys soft-skinned fruits (Hauser 2011). A serrated ovipositor allows females to deposit eggs into ripening fruits prior to harvest (Lee et al. 2011). Such spotted wing drosophila injury facilitates infestation by other *Drosophila* species (Walsh et al. 2011) and rapid decomposition of fruit. Left unmanaged, spotted wing drosophila is estimated to cause annual losses of 860 million dollars total to black berries, raspberries, and cherries in the Western United States (Hamby et al. 2012). Similar rates of loss are spreading across all U.S. small fruit production. Effective management of insect pests like *D. suzukii* requires efficient monitoring usually via trapping (Beers et al. 2011, Cha et al. 2012, Landolt et al. 2012a, Lee et al. 2012). Early and accurate detection will be the cornerstone of spotted wing drosophila management.

Understanding a fly’s basic behavioral traits can facilitate improvements in trapping. For example, spherical shape and red or dark color provided the best visual target for the apple maggot fly, *Rhagoletis pomonella *(Walsh) (Prokopy 1968). By contrast, the Mediterranean fruit fly, *Ceratitis capitata *(Wiedemann) is maximally attracted to black or yellow spheres (Nakagawa et al. 1978). Shape, color, and odor of fruits are expected to be the key determinants of ovipositional site selection by spotted wing drosophila, as they are for many other insects (Fletcher 1987, Harris and Miller 1988, Renwick and Chew 1994).

The ability of many insects to discriminate between different colors has been established beyond a doubt (Burkhardt 1964). Menzel (1979), Briscoe and Chittka (2001), and Stavenga (2002), among others, have reviewed the evolution of color vision in insects and the ability of invertebrates to perceive color, including reds. Although many moths lack photoreceptors sensitive to longer wavelengths, most day-active insects possess photoreceptors that cover the whole spectrum sensed by humans (Wakakuwa et al. 2004) and beyond into the ultraviolet (UV). As many as six different receptor types can be found in some species of Diptera (Hardie 1986).

The present investigation primarily answers the question of what type of visual cue best promotes spotted wing drosophila alightment on a small sticky surface with the ultimate aim of optimizing monitoring trap color. This is our first step in a systematic investigation of the optimal sensory parameters for spotted wing drosophila trapping. Here, the color of a visual target of arbitrarily chosen shape emitting no host odor was varied with the goal of establishing the most and the least effective colors for promoting alightment on targets at close range in the laboratory. The hypotheses tested were: 1) certain colors strongly promote spotted wing drosophila alightment, while others do not, 2) spotted wing drosophila responds differently to visual targets varying in color (reflected wavelength composition) and not just brightness of the corresponding black and white image, and 3) certain colors are preferred irrespective of whether the background shifts from light to dark.

## Materials and Methods

### 

#### *D. suzukii* Colony

Only lab-reared spotted wing drosophila were used in these experiments. Our subcolony was split from a large colony maintained at the Trevor Nichols Research Center of Michigan State University (6237 124th Avenue, Fennville, MI 49408), arising from stock field-collected on the grounds of this fruit research station. Our colony was maintained on the *D. suzukii* solid food diet of Dalton et al. (2011) in 50-ml polystyrene vials (Genesee Scientific, San Diego, CA) and held in a growth chamber at 24°C, 45% relative humidity (RH), and a photoperiod of 16:8 (L:D) h. Adults used in the experiments were 1–2 wk posteclosion. All flies were removed from their vials containing the solid food diet, lightly anesthetized with CO_2_, sorted by sex, and counted prior to the experiment. The flies entered the experiment within 1 h of being removed from the vials containing the solid food. They were not provisioned with food or water while in the bioassay cages so as to promote responsiveness to visual targets. The life span of such flies rarely exceeded 2 d under the bioassay conditions.

#### Experimental Protocols

For all experiments, 5-cm-diameter paper disks were printed on white office paper using an HP Color LaserJet CP4025 laser ink-jet printer. Disks were then glued to a second identical disk to create a double-sided colored disk. Coloration was designated according to the calibration specifications in Microsoft PowerPoint 2010 Version 14.0 (Table 1). Both sides of a disk were coated with transparent Tangle-Trap glue (Tanglefoot Company, Grand Rapids, MI) and then suspended 15 cm below the ceiling of a 60- by 60- by 60-cm white insect cage (BugDorm-2120, MegaView Science Education Services, Taichung, Taiwan). The cages were placed in four rows of three cages in a room with a 2.5-m ceiling held at 22°C and ∼63% RH. Full spectrum lighting for insects (Shields 1989) was provided by four fluorescent tubes (Lumichrome F40W 1 × C 5000 k, Lumiram Germany) affixed 18 cm from the room ceiling, and four LED fixtures (Smart Electrician, Intertek) each emitting 1,050 lumens. The distance between the lights and the sticky disks was ∼1 m. All lights were installed on timers to yield a common photoperiod of 16:8 (L:D) h. Flies were released at the bottom center of the cage at ∼1100 hours, and each test ran for only 24 h. Unless otherwise specified, the experimental design was randomized complete block, i.e., one replicate of each treatment and condition was present for a given block, and such blocks were accumulated across time, always using a fresh batch of flies. Assignment of treatments to cages (no-choice tests) or positions of treatments within a cage (choice tests) was always randomized, but with the restriction that treatments could rarely occur in the same position twice. Unless otherwise stated, count data required no transformations and were analyzed by multi-way ANOVA in JMP 10 (SAS Institute, Cary, NC, 2012); means were separated by Tukey’s HSD tests. The statistical power inherent in these experiments was deemed too low for meaningful specification of interactions. These experiments were conducted from December 2014 to May 2015. A weakness of this analysis of choice-test results was our lack of knowledge about whether or not treatments exerted their effects independently. But, comparing choice and no-choice outcomes was helpful in identifying treatment dependencies within the choice tests.

**Table 1.** Microsoft PowerPoint settings used to generate colored disks

Color output	Numerical value under Microsoft PowerPoint settings					
	RGB			HSL		
	Red	Green	Blue	Hue	Saturation	Luminosity
Red	255	0	0	0	255	128
Orange	255	102	0	17	255	128
Yellow	255	255	0	42	255	128
Green	0	128	0	85	255	64
Blue	0	0	255	170	255	128
Purple	102	0	255	213	255	51
White	255	255	255	170	0	255
Black	0	0	0	170	0	0

#### Experiment 1: Choice Tests of Colored Disks

*D.* *suzukii *response to various colors was first examined by a choice test using white cages held in a room with white walls and ceiling. The colors used were red, orange, yellow, green, blue, purple, white, and black. Additionally, a checkered black and white disk was created using the pattern-fill option in Microsoft PowerPoint; each square measured 1 mm on a side. A clear disk was fashioned from a clear 3M acetate sheet (3M Clear Gloss Acetate Label Product FA01B). One disk of each color was suspended from the top of the cage in a 25-cm-diameter circle of evenly spaced disks separated by ∼5 cm. One hundred flies were released per cage (50:50 M:F). Eight blocks were accumulated through time.

#### Experiment 2: No-Choice Tests of Colored Disks

No-choice tests were conducted by suspending a single disk of a particular color in the center of a cage 15 cm from the ceiling. Colors used were red, orange, yellow, green, blue, purple, white, black, and a checkered black and white disk. Fifty flies were released per cage (25:25 M:F). Eight replicates were accumulated through time.

#### Experiment 3: Response to Colored Disks by Gender

Experiments 1 and 2 were conducted without regard to the gender of responders. To test whether male and female *D. suzukii *responded differently to colors, a no-choice experiment was set up as per Experiment 2, but using only black, purple, red, yellow, blue, and white. One hundred males and one hundred females were now released into individual cages, each receiving a particular color. Six blocks were accumulated through time. The data were analyzed by three-way ANOVA.

#### Experiment 4: Response to Color Versus Equivalent Grayscale

Claims that an insect perceives and responds to color require evidence that the equivalent response cannot be obtained by presenting just the grayscale (brightness) equivalent of a given color (Burkhardt 1964). Accordingly, we presented spotted wing drosophila with yellow and red disks alongside those of corresponding grayscale created by printing a “color” using the grayscale setting in the printer preferences. Each cage had one such pairing of disks. One hundred flies were released into each cage (50:50 M:F). We accumulated 12 replicates for each pairing. Data were analyzed using a Student’s paired *t*-test (JMP 10). These count data required transformation to $\sqrt{x+0.5}$ to establish normality.

#### Experiment 5: Robustness of Color Responses in Black Cages

Response to color was examined in a black cage to test if there were any differences in color response due to background against which targets were presented. Black fabric was wrapped around the outside of a cage made of black netting. The top of the cage was left open and a light source mounted below a black ceiling. To rule out that failure to discriminate among colors might be explained simply by dimness, overall light intensity was increased to the level of the white-background experiments by adding an additional row of fluorescent lighting to compensate for the greater absorbance of the black background. Choice tests were conducted by suspending one disk of each color from the top of the cage as per Experiment 1. Disks were red, purple, black, blue, yellow, and white. Fifty flies were released into each cage (25:25 M:F). Eight replicates were accumulated through time. These count data required transformation to $\sqrt{x+0.5}$ to establish normality.

#### Experiment 6: Choice Tests of Fluorescent Disks

Response to various fluorescent colors was first quantified by a choice test (as per Experiment 1) using 5-cm-diameter white paper disks painted with “Americanca Neons” (DecoArt, Stanford, KY) fluorescent acrylic pigments: Fiery Red (DHS4), Thermal Green (DHS5), Sizzling Pink (DHS3), Electric Blue (DHS6), Torrid Orange (DHS2), and Scorching Yellow (DHS1). The paint dried at room temperature for 24 h and then was baked in an oven for 1 h at 50°C to further remove volatiles. One hundred flies were released into each cage (50:50 M:F). Eight replicates were accumulated through time.

#### Experiment 7: Choice Tests of Fluorescent Red Versus Nonfluorescent Red

A two-choice test was conducted to quantify rates of alightment of *D. suzukii *to nonfluorescent red and fluorescent red color. One fluorescent red disk was created by painting a 5-diameter disk with Fiery Red neon paint as per Experiment 6, and one regular red disk was created as per Experiment 1. One disk of each pigment was hung ∼10 cm apart and 15 cm from the ceiling of the cage. One hundred flies were released into each cage (50:50 M:F). Eight replicates were accumulated through time. Data were analyzed using a Student’s paired *t*-test (JMP 10).

#### Experiment 8: No-Choice Tests of Fluorescent Red Versus Nonfluorescent Red

Because fluorescent paint might activate flies from greater distances, no-choice tests were conducted to detect possible differences between choice and no-choice outcomes. One disk of either fluorescent red or nonfluorescent red was suspended centrally 15 cm below the cage ceiling. Fifty flies were released into each cage (25:25 M:F). Fourteen replicates were accumulated through time. Data were analyzed using a Student’s paired *t*-test (JMP 10). To establish normality, count data required transformation to $\sqrt{x+0.5}$.

## Results

### 

#### Experiment 1: Choice Tests of Colored Disks

**Fig. 1.**
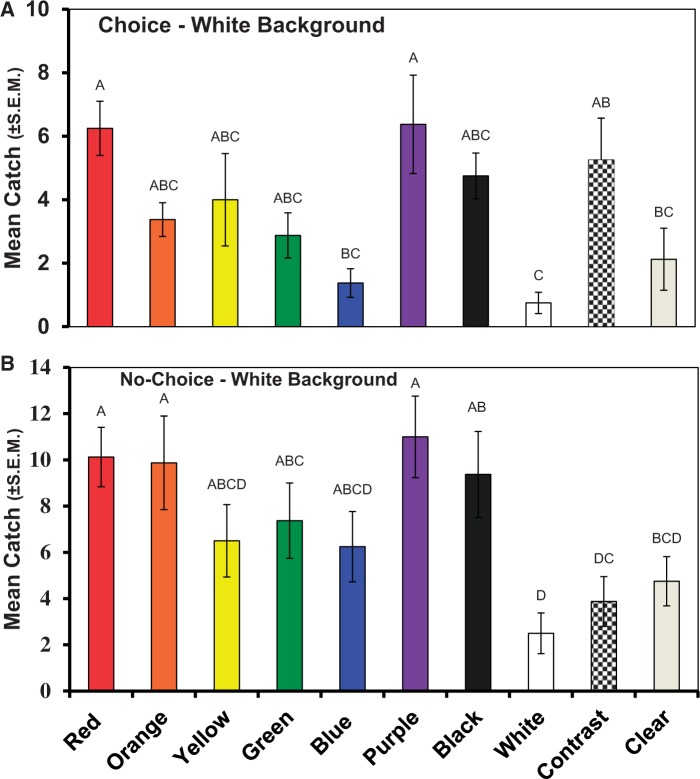
Numbers of *D. suzukii* captured on colored disks in a choice test (A), and no-choice test (B). Bars topped with a common letter within a given panel do not differ significantly at the 0.05 level. Vertical lines indicate SEM.

About 40% of the flies released in this choice experiment were recovered from the disks, suggesting that the collective set of disks near the cage ceiling stimulated many flies to visit. Numerically, white disks captured the fewest spotted wing drosophila (Fig. 1). Significantly more flies landed on red, purple, and checkered disks than on white disks (*F*_16,79_ = 3.51, *P* = 0.0002). The highest mean spotted wing drosophila captures occurred on red and purple disks; however, preference for these colors was not significantly different from many of the other colors (Fig. 1).

#### Experiment 2: No-Choice Tests of Colored Disks

**Fig. 2.**
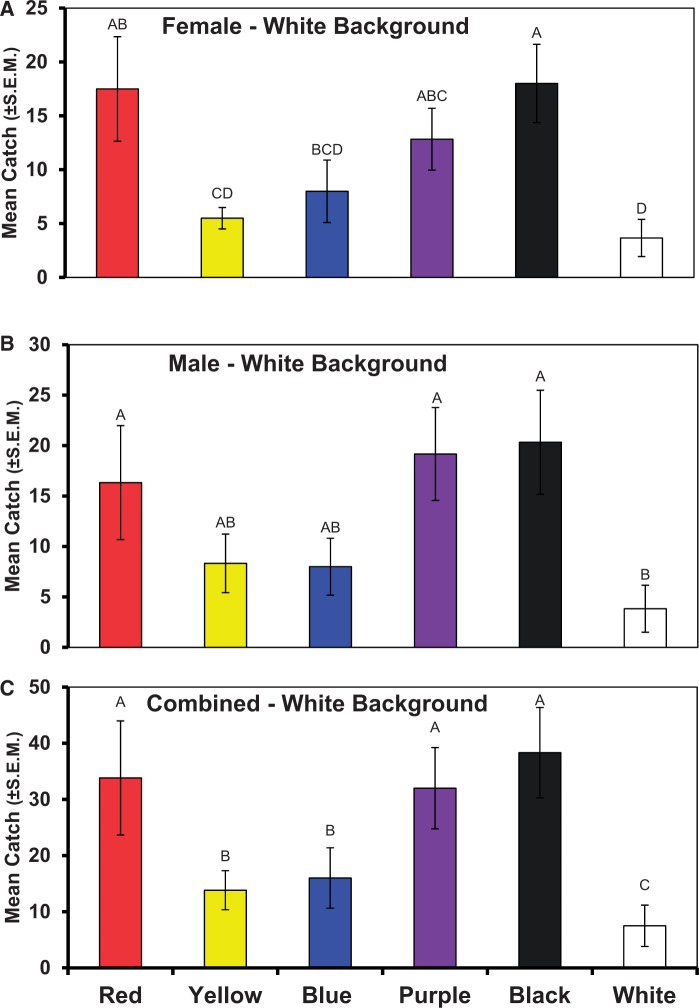
Numbers of *D. suzukii *female (A), male (B), and combined genders (C) captured on colored disks presented as a choice test. Bars topped with a common letter within a panel do not differ significantly at the 0.05 level. Vertical lines indicate SEM.

The overall pattern of spotted wing drosophila captures in the no-choice context (Fig. 2) was similar to that for choice (Fig. 1). Significantly more flies landed on red, orange, green, purple, and black disks than on white disks (*F*_16,79_ = 8.35, *P* = 0.0001), which again captured the fewest flies. Highest mean captures occurred on purple, red, orange, and black disks. However, the differences in responsiveness to all colored disks were less pronounced in the no-choice context than in the choice context, perhaps because direct competition between colors was eliminated. All of the colored disks captured only between 14–22% of the total flies released into a cage, suggesting that the overall size of a visual target or group of targets influenced the outcomes (40% were captured by a cluster of targets). Experiments 1 and 2 confirm Hypothesis 1—targets of certain colors are preferred over others when presented against a white background.

#### Experiment 3: Response to Colored Discs by Gender

No evidence was found for a gender effect in this six-choice test (*F*_5,25_ = 0.50, *P* = 0.4821). The pattern in response to colors seen above and Hypothesis 1 were further confirmed here.

#### Experiment 4: Response to Color Versus Equivalent Grayscale

Mean spotted wing drosophila capture rates on a red disk versus one of the equivalent grayscale were 12.8 ± 2.2 versus 5.4 ± 0.7, respectively (difference significant at *P* = 0.0013). Capture rates for a yellow disk versus one of the equivalent grayscale were 7.0 ± 0.8 versus 3.8 ± 0.8, respectively (difference significant at *P* = 0.0006). Thus, Hypothesis 2 (that spotted wing drosophila perceives color) is confirmed. This conclusion also held when the data were analyzed separately by gender.

#### Experiment 5: Robustness of Color Responses in Black Cages

**Fig. 3.**
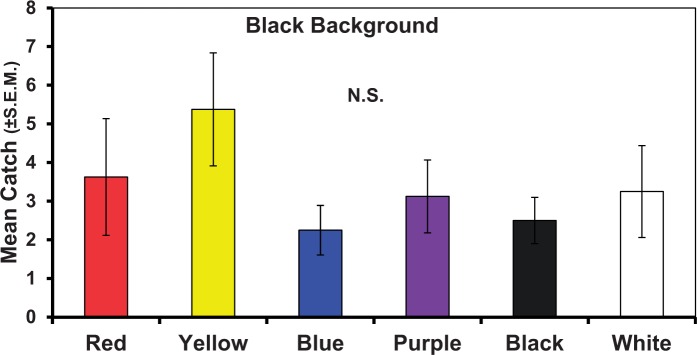
Numbers of *D. suzukii *captured on colored disks presented as a choice test with a black cage background. Bars topped with a common letter within a panel do not differ significantly at the 0.05 level. Vertical lines indicate SEM.

Against a black background, red and yellow disks captured the most spotted wing drosophila, while blue disks captured the fewest (Fig. 3). However, none of the colored disks captured significantly more spotted wing drosophila than any other (*F*_5,47_ = 1.71, *P* = 0.1543). The pattern of response to the colored disks did not follow the pattern seen in previous experiments. Experiment 5 refuted Hypothesis 3—certain colors will be preferred irrespective of whether the background shifts from light to dark.

#### Experiment 6: Choice Tests of Fluorescent Disks

**Fig. 4.**
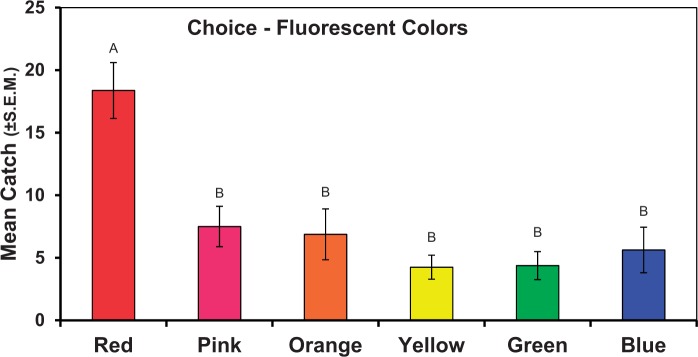
Numbers of *D. suzukii *captured on fluorescent colored disks presented as a choice test. Bars topped with a common letter within a panel do not differ significantly at the 0.05 level. Vertical lines indicate SEM.

About 47% of the flies released were recovered from the collective set of disks. Of these, 18% were captured on the fluorescent red disk, whereas the other colors each captured 6% or fewer. Numerically, fluorescent green and fluorescent yellow captured the fewest flies. Significantly more flies landed on fluorescent red disks (*F*_5,42_ = 6.53, *P = *0.0001) than all other florescent disk colors (Fig. 4). The pattern in response to colors seen in Experiment 1 and Hypothesis 1 were further confirmed here.

#### Experiment 7: Choice Tests of Fluorescent Red Versus Nonfluorescent Red

Sticky disks painted fluorescent and nonfluorescent red caught equivalently (11.8 ± 2.5 versus 10.9 ± 1.7); *t* (14) = −0.28595, *P* = 0.7797.

#### Experiment 8: No-Choice Tests of Fluorescent Red Versus Nonfluorescent Red

However, the no-choice test revealed a significant difference in the alightment rates for fluorescent red disks and nonfluorescent red disks (11.2 ± 1.1 versus 7.5 ± 0.9); *t* (26) = 2.85, *P* = 0.0085. Thus, fluorescent red emerged as the pigment most strongly promoting spotted wing drosophila visitation and alightment.

## Discussion

This study has added to the basic understanding of spotted wing drosophila behavior by proving that both genders: 1) are capable of perceiving color, and 2) respond identically to the range of colors presented either in the choice or the no-choice test. Such congruence is fortunate because a trap presenting optimal color cues for one gender will be also optimized for the other. Such an outcome cannot be taken for granted. For example, Katsoyannos and Kouloussis (2001) found that the optimal color for capturing olive fruit fly males was orange, while that for females was red. Further support that spotted wing drosophila traps will not need to be tuned by gender comes from the finding of Landolt et al. (2012b) that catch patterns across a range of odors was identical for males versus females. Similar response across both color and odor cues suggests that both genders benefit equally by responding to the same cues that might signal equally beneficial food resources. Alternatively, male responses may have been selected to match those of females so as to optimize mate finding. Many drosophilids are known to mate at ovipositional sites (Spieth 1974, Markow and Grady 2008).

Receptors maximally absorbing in the red (∼650 nm) are documented to have appeared multiple times independently in the Odonata, the Hymenoptera, and the Coleoptera (Briscoe and Chittka 2001). The spectral range covered by a given red receptor can vary even at the species level (Briscoe and Chittka 2001). For example, *Pieris rapae crucivora* has a unique visual pigment expressed in the red and another in the deep red (Wakakuwa et al. 2004). Such differences can also influence foraging behaviors. For example, an island population of *Bombus terrestis *responded significantly more strongly to red artificial flowers than did several mainland populations (Skorupski et al. 2007). Likewise, spotted wing drosophila responded strongly to red disks suggesting they are endowed with long-wavelength receptors whose inputs they respond to more strongly than many other wavelengths.

This study also has practical significance. We postulate that red, and particularly fluorescent red, will be the best pigment for monitoring traps. While no difference between red and fluorescent red was measured in the choice test, a clear and significant difference occurred in the no-choice test. Perhaps the fluorescent red disk was better at attracting spotted wing drosophila from a distance, but once nearby, flies alighted on any red object. Additionally, purple and black strongly promote spotted wing drosophila alightment (Figs. 1 and 2). This is not surprising given that host fruits for spotted wing drosophila larvae span red (strawberries, cherries, and raspberries), purple (blueberries), and black (blackberries).

The current study provides a foundation for further research addressing questions like: 1) what is the optimal shape for visual targets having a consistent color?, 2) does odor influence preference for color and shape?, 3) do the cues originating across respective sensory modalities interact additively or synergistically?, and 4) do natural largely green backgrounds and contrast influence preference for color?
